# Gut Mycobiome in Patients With Chronic Kidney Disease Was Altered and Associated With Immunological Profiles

**DOI:** 10.3389/fimmu.2022.843695

**Published:** 2022-06-16

**Authors:** Jialin Hu, Shichao Wei, Yifeng Gu, Yang Wang, Yangkun Feng, Jiayi Sheng, Lei Hu, Chaoqun Gu, Peng Jiang, Yu Tian, Wei Guo, Longxian Lv, Fengping Liu, Yeqing Zou, Feng Yan, Ninghan Feng

**Affiliations:** ^1^ Department of Urology, Affiliated Wuxi No.2 Hospital, Nantong University, Wuxi, China; ^2^ Collaborative Innovation Center for Diagnosis and Treatment of Infectious Diseases, State Key Laboratory for Diagnosis and Treatment of Infectious Diseases, The First Affiliated Hospital, School of Medicine, Zhejiang University, Hangzhou, China; ^3^ School of Medicine, Nantong University, Nantong, China; ^4^ Department of Urology, Affiliated Wuxi No.2 Hospital, Nanjing Medical University, Wuxi, China; ^5^ Department of Nephrology, Affiliated Wuxi No.2 Hospital, Nanjing Medical University, Wuxi, China; ^6^ Wuxi School of Medicine, Jiangnan University, Wuxi, China; ^7^ School of Basic Medicine, Jiangsu Vocational College of Medicine, Yancheng, China

**Keywords:** *Candida*, chronic kidney disease, microbial dysbiosis, mycobiome, immunity disorder, *Saccharomyces*

## Abstract

**Objectives:**

Mounting evidence suggests that bacterial dysbiosis and immunity disorder are associated with patients with chronic kidney disease (CKD), but the mycobiome is beginning to gain recognition as a fundamental part of our microbiome. We aim to characterize the profile of the mycobiome in the gut of CKD patients and its correlation to serum immunological profiles.

**Methods and materials:**

Ninety-two CKD patients and sex–age–body mass index (BMI)–matched healthy controls (HCs) were recruited. Fresh samples were collected using sterile containers. ITS transcribed spacer ribosomal RNA gene sequencing was performed on the samples. An immunoturbidimetric test was used to assess the serum levels of immunological features.

**Results:**

The CKD cohort displayed a different microbial community from that in the HC cohort according to principal coordinate analysis (PCoA). (*P*=0.001). The comparison of the two cohorts showed that the CKD cohort had significantly higher gut microbial richness and diversity (*P*<0.05). The CKD cohort had lower abundances of *Candida*, *Bjerkandera*, *Rhodotorula*, and *Ganoderma* compared to the HC cohort, while it had higher *Saccharomyces* (*P*<0.05). However, the microbial community alteration was inconsistent with the severity of kidney damage in patients, as only patients in CKD stage 1~3 had differed microbial community concerning for HCs based on PCoA (*P*<0.05). The serum concentration of the kappa light chain in CKD patients was positively associated with *Saccharomyces*, whereas the it was negatively associated with *Ganoderma* (*P*<0.05).

**Conclusions:**

Not only was gut mycobiome dysbiosis observed in CKD patients, but the dysbiosis was also associated with the immunological disorder. These findings suggest that therapeutic strategies targeting gut mycobiome might be effective.

## Introduction

Chronic kidney disease (CKD) is a kidney impairment that is irreversible and ultimately leads to end-stage renal disease and renal replacement therapy (e.g., transplantation or dialysis). Globally, 1.2 million people died from CKD in 2017, and the all-age mortality rate from CKD increased by 41.5% between 1990 and 2017 (1). Although the fact of the climbing incidence of CKD has been recognized as a public health problem, its etiologies are not currently clear. As the management of CKD includes treating the underlying etiologies, it is urgent to explore the potential pathology associated with the disease.

It is well established that CKD is associated with bacterial dysbiosis in the gut (2–8). The existence of gut microbiome alterations such as a reduction in bacterial diversity has been related to CKD (3, 4, 6, 7). Moreover, several potential pathogenic bacteria, such as Enterobacteriaceae, *Escherichia-Shigella*, *Klebsiella*, and *Pseudomonas* increased in CKD patients (3, 6, 7), while bacteria with probiotic features, including *Akkermansia*, *Bifidobacterium*, *Blautia*, *Faecalibacterium*, *Prausnitzii*, Lactobacillaceae, and *Roseburia*, depleted (3–5, 7).

Kidneys contribute to immune homeostasis, and the components of the immune system mediate renal disease and play a central role in the progression of CKD (9). For instance, serum complement 3 (C3), as partially originated from the kidney, was not only increased in CKD patients but also associated with a decreased estimated glomerular filtration rate (eGFR) (10). Additionally, an elevated serum C-reactive protein (CRP) level has been linked to CKD (11–13).

The kidneys are frequently targeted by pathogenic immune responses against renal autoantigens or by local manifestations of systemic autoimmunity (14). The interaction between the microbiome and immunity in health and disease might drive the pathogenesis of renal disorders (15). For example, our previous study demonstrated that the depletion of Actinobacteria and *Bifidobacterium* in CKD patients predicted the serum high level of free light chain lambda (FLC λ) (3).

Invasive fungal infections are a major challenge in the management of patients with renal dysfunction (16). It is reported that fungal infections complicate the course of 4%–7% of CKD patients, with a mortality of over 65% (17). Traditionally, fungi have been considered pathogens that commonly affect any organs of the body, including the colon. However, beyond the bacterial community, the human gut also harbors a large number of fungi (18). Gut fungi are causally implicated in microbiome assembly and immune development. Accumulating findings highlighted that the human mycobiome can strongly influence the host immune system (19, 20).

Given that CKD patients are usually complicated with fungal infection (16), and persistent immunity disorder is a common feature of CKD and inflammation is the body’s immune system response to an irritant (including bacteria, viruses, or fungi) (21, 22), we hypothesize that the gut mycobiome of individuals with and without CKD differs, and the differences correlate with their renal damage severity, along with patients’ immunological characteristics.

## Methods and Materials

### Participant Recruitment

The ethics committee of the Affiliated Wuxi No.2 Hospital of Nanjing Medical University approved this study (Ref. 2018051). Informed consent was provided by all subjects before their inclusion in the study. As sex, age, and BMI are important variables affecting the gut microbiome (23–25), we recruited ninety-two CKD patients and sex–age–BMI–matched healthy controls (HCs) were recruited from December 2018 to February 2020. The inclusion and exclusion criteria were based on our recent study on the bacterial microbiome in CKD patients (3). The diagnostic criteria for CKD include a decreased eGFR [<60 ml/min/1.73 m^2^) or an evidence of kidney damage such as albuminuria (albumin excretion rate ≥30 mg/24 h; urinary albumin– creatinine ratio (UACR) ≥30 mg/g], urine sediment abnormalities, electrolytes, and other abnormalities due to tubular disorders, abnormalities detected by histology, and structural abnormalities detected by the imaging or history of kidney transplantation (26). Patients with CKD who have never undergone hemodialysis were recruited. CKD patients were separated into five subgroups based on the stages of renal function at recruitment (27): stage 1, normal or high eGFR CKD (eGFR ≥ 90 ml/min/1.73m^2^); stage 2, mild CKD (eGFR ≥ 60~89 ml/min/1.73m^2^); stage 3, moderate CKD (30~59 ml/min/1.73m^2^); stage 4, severe CKD (15~29 ml/min/1.73m^2^); and stage 5, end-stage CKD (<15 ml/min/1.73m^2^). Subjects with kidney damage and positive urinary protein, or eGFR less than 90 ml/min/1.73 m^2^, current illness (including diabetes and hypertension), elevated body temperature, or white blood cell count or serum C-reactive protein were excluded from the HC cohort. In addition, participants with acute intercurrent disease and infections, diarrhea, kidney transplantation, pregnancy, and breastfeeding and those who used antibiotics, probiotics, or immunosuppressive drugs within 30 days before enrollment were excluded from the present study.

### Sample Collection and Procession

Fecal samples were collected in a sterile container by the patient. Approximately 30 mg of feces were transferred to a sterile bottle containing 500 μl lysis buffer containing Tris 0.1mol/L (pH 8.0), 2 mM Pleas, and 2% sodium dodecyl sulfate (SDS) (Guhe Health. com., Hangzhou, China) by a technician using sterile techniques and stored at −80°C until further processing.

The blood samples for detecting renal function and immunological features were collected on the day of fecal sample collection. An immunoturbidimetric test was used to assess the serum levels of immunological features on the day of sample collection (AU5400; Beckman Coulter, Pasadena, CA, USA). Information on clinical manifestations; concurrent diseases, such as diabetes, hypertension, and hyperlipidemia; and the current medication usage were assessed by reviewing clinical records, medical interviews, and face-to-face interviews. A Chinese version of the Food Frequency Questionnaire was used to assess food intake to measure the effects of confounding on the microbial community in CKD patients (28).

### Fungal DNA Extraction

The DNeasy PowerSoil Pro Kit was used to isolate fungal genomic DNA from fecal samples according to the manufacturer’s instructions in a biological safety cabinet (QIAGEN, Hilden, Germany), with additional glass-bead beating steps performed using a Mini-Beadbeater (FastPrep; Thermo Electron, Boston, Waltham MA, USA). The amount of DNA was determined using a NanoDrop ND-1000 spectrophotometer (Thermo Electron). The integrity and size of DNA were verified by electrophoresis on a 1.0% agarose gel containing 0.5 mg/ml ethidium bromide. All DNA samples were stored at −20°C prior to further analysis.

The ITS regions were amplified using ITS1F (5’-CTTGGTCATTTAGAGGAAGTAA-3’) and ITS2 (2043R; 5’-GCTGCGTTCTTCATCGATGC-3’) primers (29). All PCR reactions were performed using Phusion High-Fidelity PCR Master Mix (Thermo Scientific Inc., Waltham, MA, USA) according to the manufacturer’s protocol and approximately 50 ng of extracted DNA per reaction. Thermocycling conditions were set at 98°C for 15 s for 1 cycle, then at 98°C for 15 s, 58°C for 15 s, then 72°C for 15 s for 30 cycles, followed by a final extension at 72°C for 1 min. Negative DNA extraction samples (lysis buffer and kit reagents only) were amplified and sequenced as contamination controls. Amplified products were purified using Agencourt AMPure XP beads (1 volume; Beckman Coulter, Pasadena, CA, USA) and samples were run on a 1% agarose gel in order to size-select gel slices of approximately 430 bp. The amount of DNA was determined using a Qubit 2.0 Fluorometer (Life Technologies, Carlsbad, CA, USA). Sequencing was performed with 2 × 150 bp on a Novaseq 6000 platform (Illumina Inc., San Diego, CA, USA).

### Bioinformatic Analysis

The ITS sequence dataset was merged and demultiplexed into per-sample data using QIIME (V1.9.1) with default parameters (30). Raw sequencing reads with exact matches to the barcodes were assigned to their respective samples and identified as valid sequences. The low-quality sequences were filtered through the following criteria: the sequences of a specified length of <150 bp, sequences of average Phred scores of <20, sequences of ambiguous bases, and sequences of mononucleotide repeats of >8 bp. Paired-end reads were assembled using Vsearch (V2.4.4; -fastq_mergepairs -fastq_minovlen 0). Operational taxonomic unit (OTU) picking included dereplication (-derep_full length), cluster (-cluster_fast, -id 0.97), and the detection of chimeras (-uchime_ref). A representative sequence was selected from each OTU using default parameters. OTU taxonomic classification was conducted by Vsearch searching the representative sequences set against the UNITE 12_11 (https://unite.ut.ee) database (31).

An OTU table was further generated to record the abundance of each OTU in each sample and the taxonomy of the OTUs. A minimum library size was chosen to rarefy the OTUs in our present study, as it is critical to normalize the OTU table to eliminate any bias due to differences in the sampling sequencing depth. In addition, total sum scaling was applied to transform the OTU table into relative abundance by dividing the number of total reads of each sample. OTUs containing less than 0.01% of total sequences across all samples were discarded.

Sequence data analysis was performed using the QIIME and R packages (V3.2.0). OTU-level alpha richness and diversity indices, including Chao1, Shannon, and Simpson, were calculated using the OTU table. Beta diversity analysis was performed to investigate the structural variation of fungal communities across samples using Bray–Curtis metrics and visualized *via* principal coordinate analysis (PCoA) based on the permutational multivariate analysis of variance (PERMANOVA) calculated by the ‘adonis’ function.

### Statistical Analysis

Pearson’s chi-square or Fisher’s exact tests were used with categorical variables; Student’s t-test and ANOVA were used on normalized continuous variables and the Wilcoxon rank-sum test on non-normal continuous variables. The *P*-value was adjusted for multiple comparisons using the Benjamini–Hochberg (BH) false discovery rate (FDR). Spearman correlation analysis was performed on the abundant bacterial genera (>1% relative abundances) and immunological features that differed between the CKD and HCs.

## Results

### Demographics

We assessed the mycobiome and serum immunological features of a total of 92 CKD patients and 92 sex-age-BMI matched HCs (**Table 1** **)**. Both cohorts were 50% (n=46) women and 50% (n=46) men. The CKD cohort had higher levels of urinary protein, serum creatinine, urea nitrogen, uric acid, and eGFR (*P*<0.05 for all comparisons). Of the 92 CKD patients, the prevalence of renal function from stage 1~5 ranged from 23.91% to 17.39%. The CKD cohort had significantly elevated CRP, serum κ and λ light chain, and rheumatoid factor (**Table 2**; *P*<0.05). The medication usages of antihypertensive/glucocorticoid/hypoglycemic/hypolipidemic agents were not confounding factors of the difference of immunological features between the CKD patients and controls since the medication users did not differ in their immunological states from non-users (**Tables S1**–**S4**). As the nutrient intake was compared between the two cohorts, no significant difference was found (**Table S5**; *P*<0.05 for all nutrients); thus, they were not listed as confounding factors in the downstream analysis.

**Table 1:** Characteristics of participants.

Parameters		Value for cohort (n^a^)^b^ or statistic		*P*-value^c^
		CKD (n = 92)	HC (n = 92)	
Female sex, n (%)		46 (50.00)	46 (50.00)	1.000
Age (years)		56.85 ± 16.58	57.20 ± 17.36	0.890
Body mass index (kg/m2)		24.43 ± 2.26	24.72 ± 3.61	0.507
Duration of CKD (yrs)	≤1	55 (59.78)	NA	NA
	1 ~ 5	25 (27.17)		NA
	>5	12 (13.04)		NA
***Comorbidity***				
	Diabetes, n (%)	29 (31.52)	0 (0.00)	<0.001
	Hypertension, n (%)	60 (65.22)	0 (0.00)	<0.001
***Other-organs-involved diseases***				
	Coronary heart disease	8 (8.70)	0 (0.00)	<0.001
	Hepatitis B	10 (10.87)	0 (0.00)	<0.001
***Blood glucose***				
	Hemoglobin A_1c_ (mmol/mol)	6.11 ± 1.05	5.63 ± 0.42	<0.001
	Fasting blood glucose (mmol/L)	5.53 ± 2.27	4.90 ± 0.87	0.013
***Blood pressure***				
	Systolic pressure (mmHg)	144.04 ± 18.86	127.54 ± 9.77	<0.001
	Diastolic pressure (mmHg)	83.48 ± 18.17	79.10 ± 7.57	0.135
***Renal function***				
	Urine protein			
	Negative	34 (36.96)	92 (100.00)	<0.001
	(+-)	5 (5.43)	0 (0.00)	<0.001
	(+)	17 (18.48)	0 (0.00)	<0.001
	(++)	26 (28.26)	0 (0.00)	<0.001
	(+++)	8 (8.70)	0 (0.00)	<0.001
	Serum creatinine (μmol/L)	206.42 ± 196.92	56.37 ± 11.58	<0.001
	Blood urea nitrogen (mmol/L)	12.26 ± 10.02	5.37 ± 1.55	<0.001
	Serum uric acid (μmol/L)	429.34 ± 134.78	278.00 ± 89.87	<0.001
	Estimated glomerular filtration rate (ml/min/1.73 m2)	56.75 ± 38.92	108.82 ± 14.76	<0.001
***CKD stage***			NA	
	Normal or high eGFR CKD (eGFR ≥ 90 ml/min/1.73m^2^)	22 (23.91)		NA
	Mild CKD (eGFR ≥ 60~89 ml/min/1.73m^2^)	19 (20.65)		NA
	Moderate CKD (30~59 ml/min/1.73m^2^)	16 (17.39)		NA
	Severe CKD (15~29 ml/min/1.73m^2^)	19 (20.65)		NA
	End-stage CKD (<15 ml/min/1.73m^2^)	16 (17.39)		NA
			NA	
***Medication usage***	Antihypertensive agents	42 (45.65)		NA
	Glucocorticoid agents	8 (8.70)		NA
	Hypoglycemic agents	11 (11.96)		NA
	Hypolipidemic agents	9 (9.78)		NA

a n, number of subjects;

b Mean ± SD or n (%);

c Pearson chi-square or Fisher’s exact test was used with categorical variables and Student’s t-test on normalized continuous variables.

CKD, chronic kidney disease; NA, not applicable.

**Table 2:** Comparison of immunological status between CKD and HC cohort.

Parameters	Value for cohort (n^a^)^b^ or statistic		*P*-value^c^
	CKD (n = 92)	HC(n = 92)	
CRP (mg/L)	4.92 ± 6.45	2.53 ± 1.38	0.001
Serum κ light chain (mg/L)	8.81 ± 2.92	1.87 ± 0.33	<0.001
Serum λ light chain (mg/L)	4.92 ± 2.58	3.31 ± 1.23	<0.001
Complement C3(g/L)	0.81 ± 0.21	1.41 ± 4.38	0.199
Complement C4(g/L)	0.24 ± 0.08	0.67 ± 4.45	0.355
Immunoglobulin A (g/L)	2.61 ± 1.07	2.47 ± 1.02	0.356
Immunoglobulin G (g/L)	10.95 ± 3.62	11.53 ± 1.86	0.172
Immunoglobulin M (g/L)	1.16 ± 0.91	1.12 ± 0.67	0.712
Antistreptolysin-O (U/ml)	49.91 ± 49.08	58.66 ± 38.71	0.160
Rheumatoid factor			0.023
Positive	5 (5.43)	0 (0.00)	
Negative	87 (94.57)	92 (100.00)	
Rheumatoid factor (U/ml)^d^	1.78 ± 13.26	NA	NA

a n, number of subjects;

b Mean ± SD or n (%);

c Pearson chi-square or Fisher’s exact test was used with categorical variables, Student’s t-test on normalized continuous variables, and Wilcoxon rank-sum test on non-normal continuous variables.

CKD, chronic kidney disease; CRP, C-reactive protein; NA, not applicable.

### Gut Mycobiome Was Altered in CKD Patients

**Figure 1:**
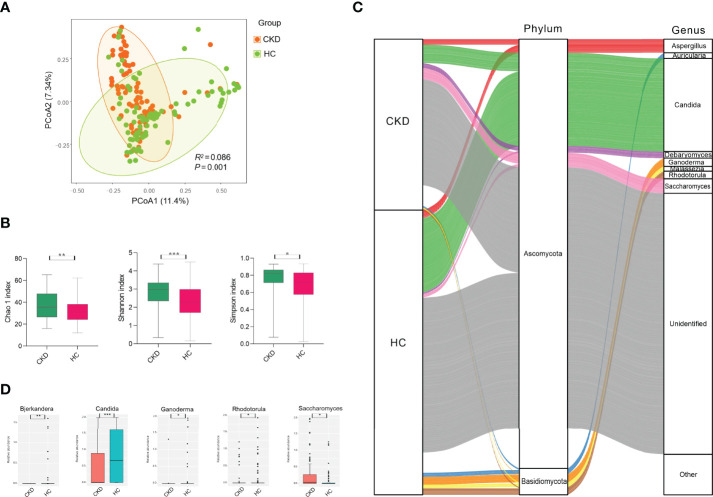
Microbial community, diversity, composition, and differed genera in the cohorts of CKD and HC. **(A)** PCoA based on Bray–Curtis distances at the OTU level showed different microbial compositions between groups of CKD patients and HCs (*P* < 0.05). Permutational multivariate analysis of variance (PERMANOVA) was performed for statistical comparisons of samples in the two cohorts. *P*-value was adjusted by the Benjamini–Hochberg FDR. **(B)** Bacterial richness and diversity measured by Chao1, Shannon, and Simpson were calculated at the microbial OTU level. The CKD patients had significantly higher levels of bacterial richness and diversity. The Wilcoxon rank-sum test was performed and adjusted by the Benjamini–Hochberg FDR. ** indicates *P* < 0.01. **(C)** Microbial profile at the phylum and genus levels. Sankey plot representing the overall gut mycobiome composition and corresponding abundance area for CKD patients and HCs. The taxonomic classification levels of phylum and genus are displayed. The top ten most abundant genera and their affiliated phyla are shown in the Sankey plot. **(D)** Microbial genera that were differentially abundant between CKD patients and HCs. Only the genera with above 1% are displayed. *P*-value was calculated using the Wilcoxon rank-sum test and adjusted by the Benjamini and Hochberg FDR. *, **, and *** indicate *P* < 0.05, *P* < 0.01, and *P* < 0.001, respectively.

To test whether the bladder microbiome differs between CKD patients and HCs, we first assessed the microbial community structure using all OTUs presenting in each sample. The PCoA of Bray–Curtis dissimilarities revealed a differential clustering between the CKD patients and HCs (**Figure 1A**; *R^2 ^=*0.086, *P*=0.001), reflecting a dysbiosis fungal gut microbiome in CKD patients. As only the participants in the CKD cohort were being administered medications at recruitment, we compared the CKD patients who were being administered antihypertensive/glucocorticoid/hypoglycemic/hypolipidemic agents and non-users using PCoA to clarify the confounding effects of medication usage. In addition, no significant differences were found between the users and non-users (**Figures S1A, B**; *P*>0.05), indicating that the abovementioned medication administrations were not confounding factors when we compare the microbiome between CKD patients and controls. As the mounting of studies demonstrated that gender plays a role in the human bacterial microbiome in the gut (32–34), we compared the bacterial community between the male and female subjects from either the CKD group and controls. However, we noticed that there were no differences between the men and women in both groups (**Figure S2**; *P*>0.05).

When the bacterial richness and diversity were assessed, the CKD patients demonstrated significantly higher levels of Chao1, Shannon, and Simpson indices compared to those in the HC cohort (**Figure 1B** **;** *P*<0.05).

As shown in **Figure 1C**, either the CKD cohort or HC cohort was dominated by Ascomycota (28.39% vs. 35.64%) and Basidiomycota (11.61% vs. 13.04%) at the phylum level, but they did not show significant differences (*P*>0.05). At the genus level, four identified fungi were predominant (above 1% abundance) in the CKD cohort, including *Candida* (9.95%), *Saccharomyces* (5.60%), *Aspergillus* (2.94%), and *Debaryomyces* (2.63%). In addition, we noticed that there were more fungal genera with an abundance above 1% in the HC cohort, such as *Candida* (24.21%), *Aspergillus* (2.65%), *Ganoderma* (2.52%), *Rhodotorula* (2.08%), *Auricularia* (1.33%), *Malassezia* (1.20%), and *Saccharomyces* (1.12%).

When the fungal genus with an abundance above 1% was compared, the CKD cohort had significantly lower abundance of *Candida*, *Bjerkandera*, *Rhodotorula*, and *Ganoderma* compared to the HC cohort, while it had a higher level of *Saccharomyces* (**Figure 1D**; *P*<0.05). At the fungal species level, we did not observe any species displaying a significant difference between the CKD patients and healthy subjects (*P*>0.05).

### Gut Mycobiome Fluctuated With CKD Stages

**Figure 2:**
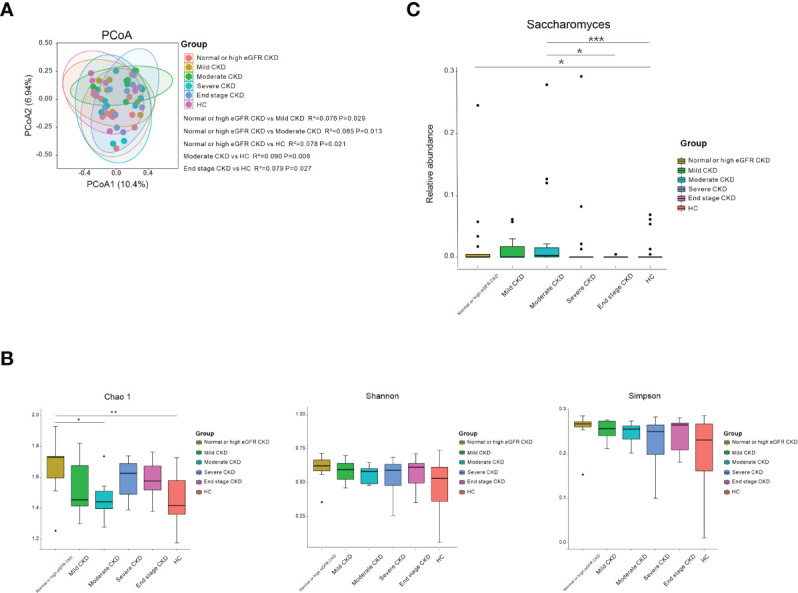
Microbial community, diversity, composition, and Saccharomyces in the subgroups according to CKD stages and HC. **(A)** PCoA based on Bray–Curtis distances at the OTU level showed different microbial compositions between the subgroups of patients’ renal function damage and HCs. Permutational multivariate analysis of variance (PERMANOVA) was performed for statistical comparisons of samples in the two cohorts. Patients with normal- or high-eGFR CKD/moderate CKD/end-stage CKD showed different microbial communities compared to HCs/mild CKD/moderate CKD (*P* < 0.05). *P*-value was adjusted by the Benjamini and Hochberg FDR. **(B)** Bacterial richness and diversity measured by Chao1, Shannon, and Simpson were calculated at the microbial OTU level. Chao1 showed significantly higher in normal or high eGFR CKD in relation to HCs/moderate CKD (P < 0.05). Wilcoxon rank-sum test was performed and adjusted by the Benjamini and Hochberg FDR. * and ** indicate *P* < 0.05 and *P* < 0.01, respectively. **(C)** Comparison of the abundances of *Saccharomyes* in CKD patients from normal or high eGFR CKD to end-stage CKD and HC. *, **, and *** indicate *P* < 0.05, *P* < 0.01, and *P* < 0.001, respectively.

The CKD patients were divided into five subgroups based on CKD stages (29). Ten patients in each CKD stage and 10 healthy subjects who were individually sex–age–BMI matched were selected (**Table S6**). We noticed that the microbial community in patients with normal or high eGFR CKD/moderate CKD/end-stage CKD was significantly different from HCs; patients with normal or high eGFR CKD differed from mild/moderate CKD (**Figure 2A**; *P*<0.05). When the bacterial richness and diversity were assessed, only Chao1 showed significantly higher levels in patients with normal or high eGFR compared to that in patients with moderate CKD and HCs (**Figure 2B**; *P*<0.05). When microbial genera were compared among groups, *Saccharomyces* was significantly elevated in patients with normal or high eGFR CKD and patients with moderate CKD compared to HCs. In addition, patients with moderate CKD had significantly increased *Saccharomyces* than that in the patients with end-stage CKD (**Figure 2C**; *P*<0.05).

### Immunological Features Were Altered in CKD Patients and Associated With Gut Mycobiome

**Figure 3:**
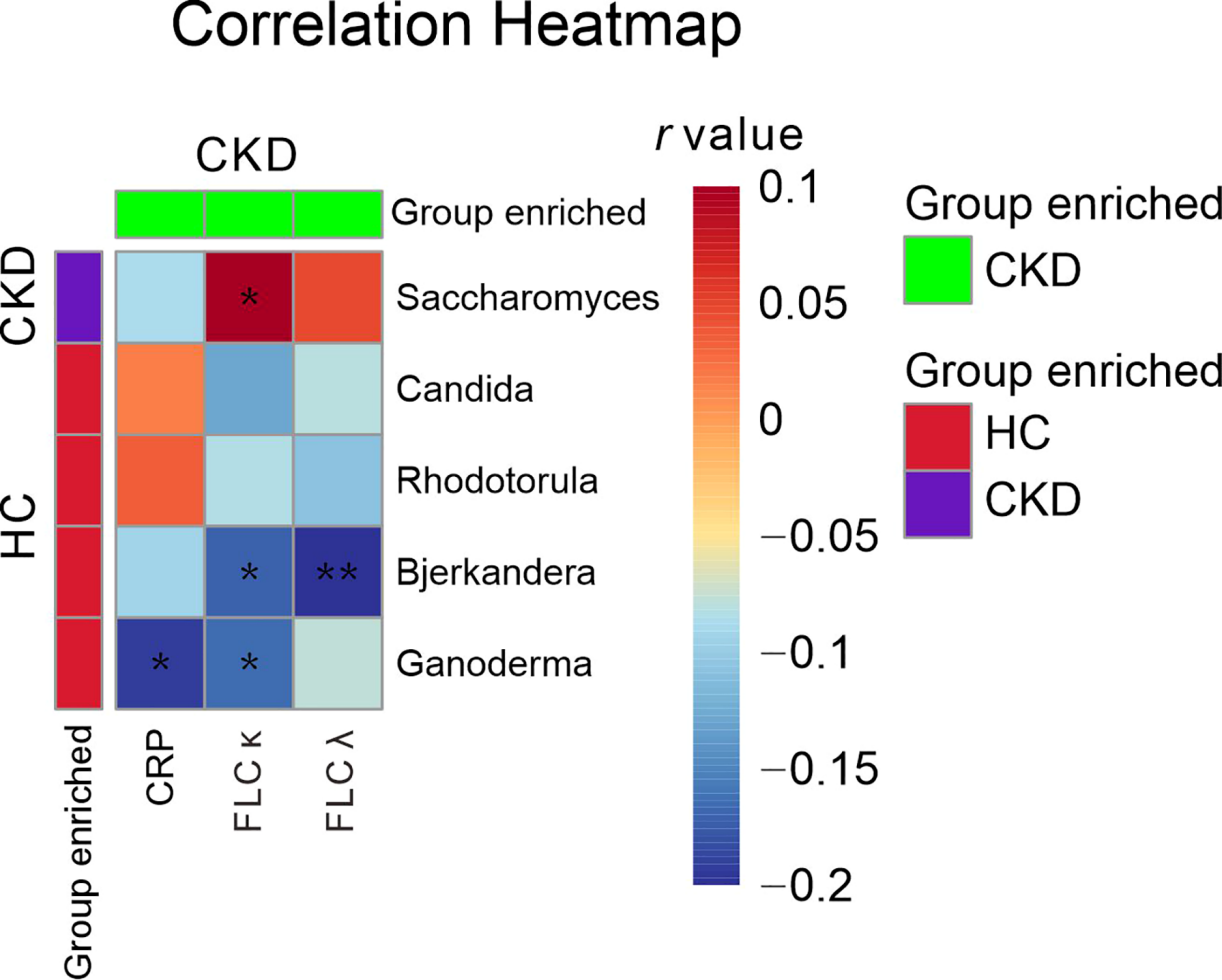
Gut mycobiome correlation to immunological profiles. Spearman correlation analysis was performed on the abundant bacterial genera (>1% relative abundances) that displayed a significant difference between CKD patients and HCs and the disease profiles that showed significant difference between CKD patients and HCs. The correlations of two variables with values of *P* < 0.05 are displayed. *, and ** indicate *P* < 0.05 and *P* < 0.01 respectively.

When the serum immunological features were compared, the CKD patients had significantly higher levels of serum CRP, FLC κ and FLC λ, and rheumatoid factor (**Table 2**; *P*<0.05). Next, we assessed the associations using Spearman correlation analysis between the immunological features (except for the rheumatoid factor) and fungal genera that were altered in the CKD cohort. We noticed that the CKD-enriched genus, *Saccharomyces*, was positively correlated to the enriched level of FLC κ in CKD patients (**Figure 3**, *P*<0.05). However, the CKD-depleted genera, *Bjerkandera* and *Ganoderma*, were negatively correlated to FLC κ. In addition, the reduction of the microbial genus, *Bjerkandera*, was negatively correlated to the enhanced expression of FLC λ in CKD patients. The decreased abundance of *Ganoderma* was also negatively correlated to the increased level of serum CRP (**Figure 3**, *P*<0.05).

As the rheumatoid factor was detectable only in 5/92 CKD patients and was not detected in any healthy subjects, we did not perform a correlation analysis between the microbial genus and the rheumatoid factor.

## Discussion

As the association of the gut bacterial microbiome with the health state has gained attention and its correlation to immunity has been extensively explored in recent years, we firstly explored the fungal profile in CKD patients’ gut and its associations with serum immunological features. Like the bacterial microbiome, the mycobiome is also strongly affected by environmental factors (35), such as dietary exposure (36). Therefore, we collected and compared the nutrient intake of participants in our present study. However, at the current sample size, we did not observe a difference in the food intake between CKD patients and HCs.

Similar to the bacterial profile in CKD patients (2–8), the fungal community was altered in patients in our present study. However, the alteration was fluctuated in various clinical CKD stages. The CKD patients with normal or high eGFR, moderate CKD, and end-stage CKD displayed a different fungal community with respect to healthy subjects, whereas the patients with mild CKD and severe CKD did not exhibit a difference from that in the HC cohort. These findings also appeared in our previous study on the bacterial microbiome in CKD patients (3). The restoring process that appeared in patients, including the fungal and bacterial microbiome, should be examined using animal models.

The CKD patients possessed higher levels of microbial richness and a diversity of mycobiome, which was dissimilar to several previous bacterial microbiome studies in CKD patients (3, 4, 6, 7). When we divided the CKD patients into five subgroups based on their clinical stages of CKD, we found that the higher bacterial richness was only observed in the stage 1 patients with respect to HCs. In addition, the stage 1 patients also demonstrated higher bacterial richness than that in stage 3 patients. Ren Z. et al. compared the bacterial diversity in the gut in CKD patients in which the patients were grouped into three subgroups, such as group A (stage 1~2 patients), group B (stage 3~4 patients), and group C (stage 5 patients) (7). Additionally, their study demonstrated that there were no significant differences among the three subgroups (7). We cannot conclude that our findings were dissimilar to the Ren Z. et al. study (7) as we used a different grouping method that might influence the findings.

Reports using next-generation sequencing have found diverse fungal communities in all sections of the human gut, consisting mainly of the phyla Ascomycota and Basidiomycota (37–39). Similarly, our present study also found that Ascomycota and Basidiomycota dominated in the participant’s gut, either CKD patients or HCs.

It is worthy to note that *Candida* was the most prevalent fungus in both CKD and HC cohorts, which was significantly depleted in the patients. A similar finding was observed in Sciavilla, P and his colleague’s study (40). When they compared the fungal microbiome using culturomics in the gut in patients with irritable bowel syndrome, *Candida* spp. exhibited a higher frequency in the patient group compared to control (40). Di Paola M also reported that patients with Crohn’s disease had higher levels of isolated *Candida* spp. compared to the healthy subjects (41). Therefore, the role of *Candida* spp. in the fungal community in the gut should be extensively explored using animal models in the future. As gut wall inflammation is the key pathology in patients with irritable bowel syndrome and Crohn’s disease (42), and it is also common in CKD patients, it is necessary to investigate the role of *Candida* spp. in the gut of CKD patients.

Previous mycobiome studies frequently reported that *Saccharomyces* was one of the most prevalent fungi in the human gut (18, 37, 39, 43, 44). Our present study also demonstrated that it was one of most abundant fungi in the participants’ gut, especially in the CKD patients. Moreover, the high level of *Saccharomyces* was responsible for the high concentration of serum FLC κ in CKD patients, as the abundance of *Saccharomyces* was positively associated with the level of FLC κ in patients. *Saccharomyces* spp. is a probiotic whose clinical efficacy, anti-inflammatory, and immunomodulatory effects are supported by previous extensive studies (45, 46). As FLCs, such as FLC κ, accumulate in the circulation, their concentrations progressively increase with renal damage in CKD patients (47).

We noticed that *Bjerkandera* was declined in CKD patients and it was negatively correlated to the levels of FLC κ and λ in patients. It is necessary to explore the immunological function of *Bjerkandera* in the human gut, as it has been rarely reported by human gut microbiome studies.

In the present study, *Ganoderma* decreased in CKD patients’ gut and it was negatively linked to the levels of CRP and FLC κ. *Ganoderma* spp., a component of edible mushroom, has potential beneficial effects. Thus, the increase of *Saccharomyces* in the patients’ gut and its positive link to FLC κ might be a protective response in CKD progression (48, 49). For example, Chang and colleagues have shown that *Ganoderma* spp. reduced obesity by modulating the gut microbiome (48). Wu et al. also demonstrated that the administration of *Ganoderma* spp. increased the abundance of beneficial bacteria in the gut of mice (49). Therefore, the decrease of *Ganoderma* in CKD patients and its negative associations with CRP and FLC κ suggest that *Ganoderma* might play a potential role of immune modulation in the onset and progression of CKD.

Renal diseases are associated with an imbalanced bacterial microbiome and the loss of immune homeostasis (2–9). Coincidentally, our present study demonstrated that dysbiosis of the gut mycobiome is evident in CKD patients and it is associated with the immunological disorders. It is especially noteworthy that *Saccharomyces* and *Ganoderma*, the components of probiotics (45), were linked with patients’ immunological profiles. For example, a previous study reported that the administration of *Saccharomyces boulardii* to patients with HIV lowered the concentrations of some gut bacterial species, such as the Clostridiaceae family, which were correlated with systemic levels of bacterial translocation and inflammation markers (45). Another study demonstrated that Reishi mushroom increased the gut Bacteroides/Firmicutes ratio and promoted the growth of anti-inflammatory and short-chain fatty acid (SCFA)–producing bacteria (50). Thus, the findings in our present study shed light on mycobiome-based therapy in CKD patients.

There are limitations to our present study. On one hand, we cannot remove the bias led by the co-occurrence of diabetes and hypertension in CKD patients as renal damage is one of the most common complications of diabetes and hypertension (51,52). On the other hand, although we compared the composition of microbiome using subgroups based on the patients’ clinical stages, the small sample size might skew the findings. A future study with a large and multicenter population is needed to confirm the association between the severity of renal damage and the mycobiome.

## Data Availability Statement

The original contributions presented in the study are publicly available. This data can be found here: https://www.ncbi.nlm.nih.gov/sra/?term=PRJNA647266.

## Ethics Statement

The studies involving human participants were reviewed and approved by the ethics committee of the Affiliated Wuxi Second Hospital of Nanjing Medical University (Ref. 2018051). The patients/participants provided their written informed consent to participate in this study.

## Author Contributions

Conceptualization: LL, FL, YZ, FY, and NF. Methodology: JH, SW, YW, YF, JS, LH, CG, PJ, YT, WG, and FY. Software: FL, JH, and WG. Validation: NF. Writing: JH, FL, YZ, and YG. Supervision: NF. Funding acquisition: NF. Project administration: FL, JH, LH, PJ, JS, CG, YF, FY, and YW. All authors contributed to the article and approved the submitted version.

## Funding

Wuxi “Taihu Talents Program” Medical and Health High-level Talents Project (THRCJH20200901); Wuxi “key medical discipline construction” Municipal Clinical Medical Center (municipal public health center) Project (LCYXZX202103).

## Conflict of Interest

The authors declare that the research was conducted in the absence of any commercial or financial relationships that could be construed as a potential conflict of interest.

## Publisher’s Note

All claims expressed in this article are solely those of the authors and do not necessarily represent those of their affiliated organizations, or those of the publisher, the editors and the reviewers. Any product that may be evaluated in this article, or claim that may be made by its manufacturer, is not guaranteed or endorsed by the publisher.

## Supplementary Material

Supplementary Figure 1 The effects of medication usageon CKD patients’ gut mycobiome. PCoA based on Bray-Curtis distances at OUT-level did not show different microbial compositions between CKD patients who took antihypertensive agent and those who had not take antihypertensive agent **(A)**; who took glucocorticoid agent and those did not take glucocorticoid agent **(B)**; who took hypoglycemic agent and those did not take hypoglycemic agent **(C)**; who took hypolipidemic agent and those did not take hypolipidemic agent **(D)**; Permutational multivariate analysis of variance (PERMANOVA) was performed for statistical comparisons of samples using different levels of hydroxychloroquine. *P*-value was adjusted by Benjamini and Hochberg false discovery rate.
