# Supplementary material for: Gut Mycobiome in Patients With Chronic Kidney Disease Was Altered and Associated With Immunological Profiles
Source: Front Immunol. 2022 Jun 16;13:843695. doi: 10.3389/fimmu.2022.843695 (PMC9245424; doi:10.3389/fimmu.2022.843695)
Supplement: Supplementary Figure 1 — The effects of medication usageon CKD patients’ gut mycobiome. PCoA based on Bray-Curtis distances at OUT-level did not show different microbial compositions between CKD patients who took antihypertensive agent and those who had not take antihypertensive agent (A); who took glucocorticoid agent and those did not take glucocorticoid agent (B); who took hypoglycemic agent and those did not take hypoglycemic agent (C); who took hypolipidemic agent and those did not take hypolipidemic agent (D); Permutational multivariate analysis of variance (PERMANOVA) was performed for statistical comparisons of samples using different levels of hydroxychloroquine. P-value was adjusted by Benjamini and Hochberg false discovery rate. [file DataSheet_1.pdf]

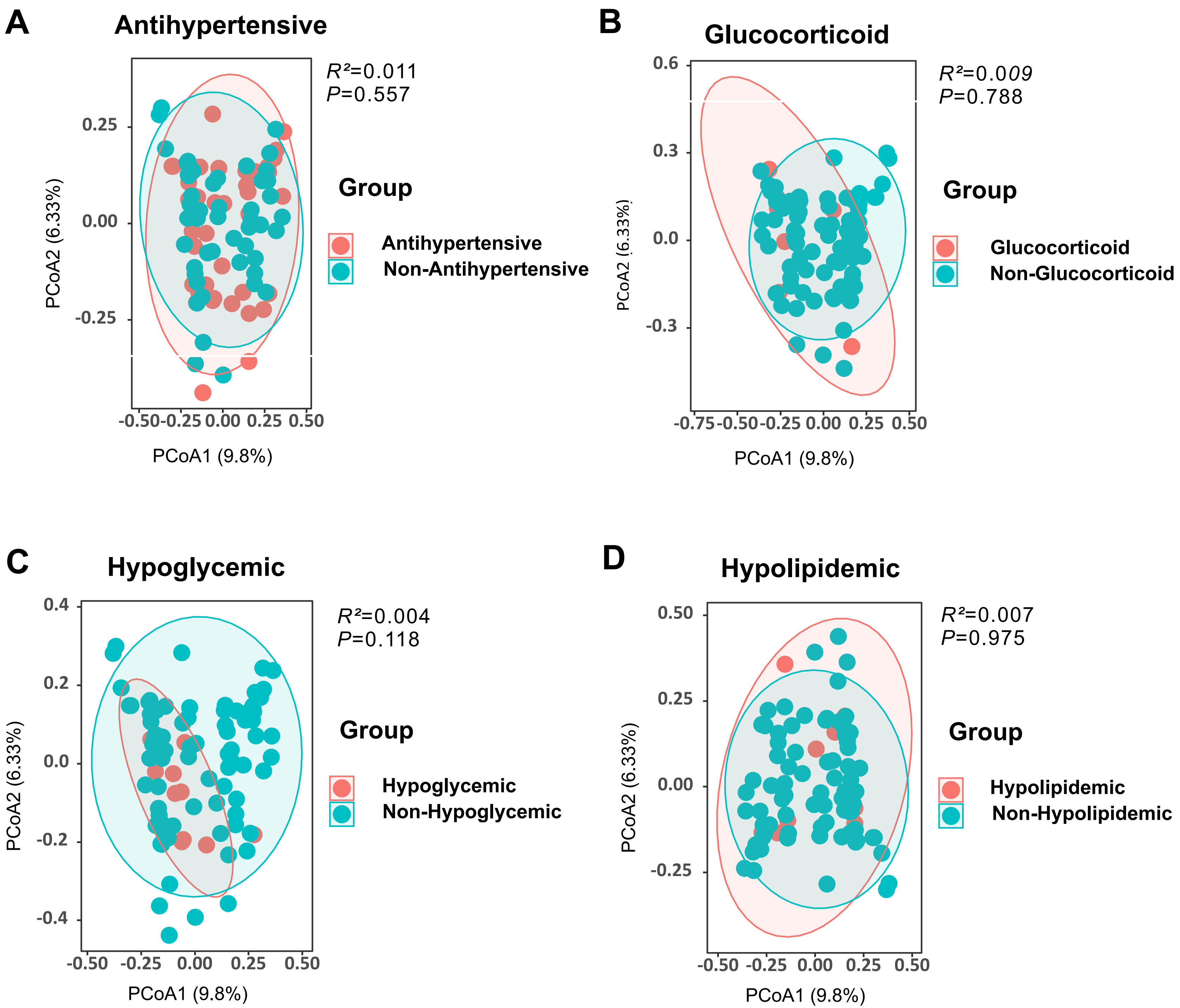
A
Antihypertensive
R^2=0.011
P=0.557
Group
Antihypertensive
Non-Antihypertensive
PCoA2 (6.33%)
PCoA1 (9.8%)
B
Glucocorticoid
R^2=0.009
P=0.788
Group
Glucocorticoid
Non-Glucocorticoid
PCoA2 (6.33%)
PCoA1 (9.8%)
C
Hypoglycemic
R^2=0.004
P=0.118
Group
Hypoglycemic
Non-Hypoglycemic
PCoA2 (6.33%)
PCoA1 (9.8%)
D
Hypolipidemic
R^2=0.007
P=0.975
Group
Hypolipidemic
Non-Hypolipidemic
PCoA2 (6.33%)
PCoA1 (9.8%)

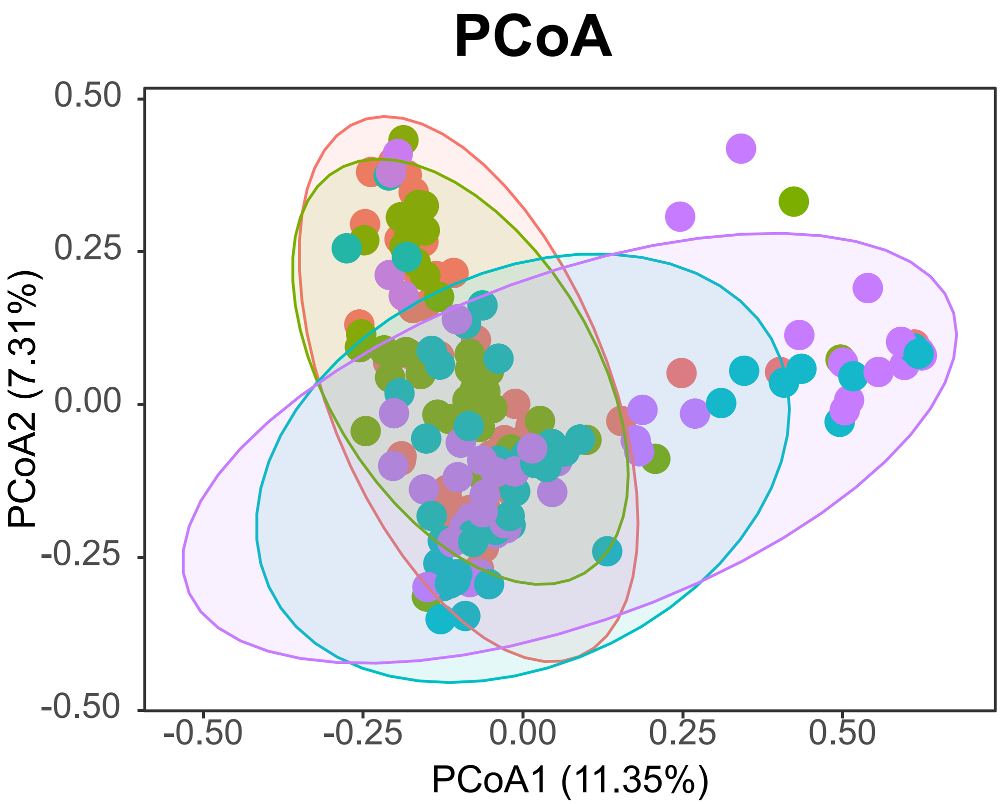

CKD-male vs CKD-female R^2=0.012 P=0.373

HC-male vs HC-female R^2=0.011 P=0.373

CKD-male vs HC-male R^2=0.030 P=0.003

CKD-female vs HC-female R^2=0.028 P=0.003

Table S1 Comparison of immunological status between antihypertensive users and non-users

Parameters	Value for cohort (n [a]) [b] or statistic		*P* value [c]
	Users (n=42)	Non-users (n=50)	
CRP (mg/L)	3.80 ± 3.12	5.82 ± 8.13	0.109
Serum κ light chain (mg/L)	9.07 ± 3.53	8.61 ± 2.34	0.451
Serum λ light chain (mg/L)	4.85 ± 1.60	4.97 ± 3.18	0.709
Completment C3（g/L）	0.81 ± 0.22	0.82 ± 0.21	0.823
Completment C4（g/L）	0.23 ± 0.07	0.24 ± 0.09	0.900
Immunoglobulin A (g/L)	2.72 ± 1.01	2.53 ± 1.10	0.397
Immunoglobulin G (g/L)	11.40 ± 4.13	10.59 ± 3.16	0.299
Immunoglobulin M (g/L)	1.18 ± 1.23	1.16 ± 0.55	0.915
Antistreptolysin-O (U/mL)	52.36 ± 57.31	47.94 ± 41.80	0.670
Rheumatoid factor			0.841
Positive	3 (7.14)	2 (4.00)	
Negative	39 (92.86)	48 (96.00)	
Rheumatoid factor (U/mL)[d]	3.13 ± 19.30	0.76 ± 41.80	0.408

[a] n, number of subjects;

[b] Mean ± SD or n (%);

[c] Pearson Chi-square or Fisher's exact test was used with categorical variables; Student's *t* test on normalized continuous variables, and Wilcoxon rank-sum test on non-normal continuous variables.

Table S2 Comparison of immunological status between glucocorticoid users and non-users

Parameters	Value for cohort (n [a]) [b] or statistic		*P* value [c]
	Users (n=8)	Non-users (n=84)	
CRP (mg/L)	9.77 ± 14.89	4.46 ± 4.94	0.348
Serum κ light chain (mg/L)	9.86 ± 6.96	8.71 ± 2.27	0.293
Serum λ light chain (mg/L)	4.85 ± 3.31	4.93 ± 2.53	0.932
Completment C3（g/L）	0.84 ± 0.16	0.81 ± 0.22	0.764
Completment C4（g/L）	0.20 ± 0.07	0.24 ± 0.08	0.243
Immunoglobulin A (g/L)	2.60 ± 1.51	2.61 ± 1.03	0.974
Immunoglobulin G (g/L)	11.39 ± 8.66	10.90 ± 2.82	0.876
Immunoglobulin M (g/L)	1.07 ± 0.31	1.17 ± 0.95	0.754
Antistreptolysin-O (U/mL)	43.20 ± 45.55	50.54 ± 49.61	0.688
Rheumatoid factor			1.000
Positive	0 (0.00)	5 (5.95)	
Negative	8 (100.00)	79 (94.05)	
Rheumatoid factor (U/mL)[d]	14.88 ± 42.07	0.47 ± 4.22	0.365

[a] n, number of subjects;

[b] Mean ± SD or n (%);

[c] Pearson Chi-square or Fisher's exact test was used with categorical variables; Student's *t* test on normalized continuous variables, and Wilcoxon rank-sum test on non-normal continuous variables.

Table S3 Comparison of immunological status between hypoglycemic users and non-users

Parameters	Value for cohort (n [a]) [b] or statistic		*P* value [c]
	Users (n=11)	Non-users (n=81)	
CRP (mg/L)	2.55 ± 2.33	5.18 ± 6.71	0.249
Serum κ light chain (mg/L)	9.02 ± 2.22	8.79 ± 2.30	0.829
Serum λ light chain (mg/L)	4.61 ± 1.15	4.95 ± 2.69	0.709
Completment C3（g/L）	0.82 ± 0.20	0.78 ± 0.30	0.626
Completment C4（g/L）	0.24 ± 0.05	0.24 ± 0.09	0.905
Immunoglobulin A (g/L)	2.64 ± 1.09	2.27 ± 0.74	0.354
Immunoglobulin G (g/L)	10.92 ± 3.67	11.25 ± 3.21	0.814
Immunoglobulin M (g/L)	1.18 ± 0.94	0.97 ± 0.44	0.518
Antistreptolysin-O (U/mL)	61.50 ± 61.13	48.65 ± 47.87	0.459
Rheumatoid factor			1.000
Positive	1 (9.01)	4 (4.94)	
Negative	10 (90.9)	77 (95.06)	
Rheumatoid factor (U/mL)[d]	0.00 ± 0.00	1.96 ± 13.90	0.693

[a] n, number of subjects;

[b] Mean ± SD or n (%);

[c] Pearson Chi-square or Fisher's exact test was used with categorical variables; Student's *t* test on normalized continuous variables, and Wilcoxon rank-sum test on non-normal continuous variables.

Table S4 Comparison of immunological status between hypolipidemic users and non-users

Parameters	Value for cohort (n [a]) [b] or statistic		P value [c]
	Users (n=9)	Non-users (n=83)	
CRP (mg/L)	2.55 ± 2.32	5.18 ± 6.71	0.249
Serum κ light chain (mg/L)	9.02 ± 2.22	8.79 ± 2.30	0.829
Serum λ light chain (mg/L)	4.61 ± 1.15	4.95 ± 2.69	0.709
Completment C3（g/L）	0.78 ± 0.30	0.78 ± 0.30	0.626
Completment C4（g/L）	0.24 ± 0.05	0.24 ± 0.09	0.905
Immunoglobulin A (g/L)	2.27 ± 0.74	2.64 ± 1.09	0.354
Immunoglobulin G (g/L)	11.24 ± 3.21	10.92 ± 3.67	0.814
Immunoglobulin M (g/L)	0.97 ± 0.44	1.18 ± 0.94	0.518
Antistreptolysin-O (U/mL)	61.50 ± 61.13	48.65 ± 47.87	0.459
Rheumatoid factor			0.987
Positive	1 (11.11)	4 (4.82)	
Negative	8 (88.89)	79 (95.18)	
Rheumatoid factor (U/mL)[d]	0.00 ± 0.00	1.96 ± 13.91	0.693

[a] n, number of subjects;

[b] Mean ± SD or n (%);

[c] Pearson Chi-square or Fisher's exact test was used with categorical variables; Student's *t* test on normalized continuous variables, and Wilcoxon rank-sum test on non-normal continuous variables.

Table S5 Comparison of nutrient intake between CKD and HC chohort

Nutrients	CKD<br>（n=92)	HC<br>(n=92)	*P* value
Energy (kcal/d)	2156.59 ± 340.39	2052.21 ± 142.25	0.383
Protein (g/d)	71.81 ± 20.42	66.06 ± 10.63	0.440
Fat (g/d)	140.49 ± 13.9	132.93 ± 7.67	0.149
Carbohydrate (g/d)	150.58 ± 63.41	145.66 ± 23.16	0.820
Fiber (g/d)	4.64 ± 2.37	4.38 ± 0.98	0.752
Vitamin A	430.13 ± 291.5	264.59 ± 151.23	0.128
Carotene	7.87 ± 2.07	10.07 ± 6.06	0.292
Retinol (μg/d）	738.08 ± 96.67	641.27 ± 122.37	0.065
Cholesterol (mg/d)	506.9 0± 167.62	375.37 ± 207.92	0.137
Vitamin B1 (mg/d)	0.53 ± 0.20	0.44 ± 0.21	0.345
Vitamin B2 (mg/d)	1.1 0± 0.54	0.89 ± 0.45	0.362
Vitamin C (mg/d)	42.19 ± 33.87	33.12 ± 15.04	0.449
Vitamin E (mg/d)	48.23 ± 3.63	40.64 ± 13.23	0.449
Calcium (mg/d)	508.80 ± 314.71	529.57 ± 321.98	0.886
Phosphorus (mg/d)	816.85 ± 194.86	803.34 ± 165.75	0.869
Potassium (mg/d)	1381.12 ± 317.48	1308.03 ± 228.69	0.562
Sodium (mg/d)	628.74 ± 245.02	1335.45 ± 1355.39	0.122
Magnesium (mg/d)	185.82 ± 35.57	221.66 ± 73.58	0.182
Iron (mg/d)	20.76 ± 12.06	16.69 ± 4.79	0.334
Zinc (mg/d)	15.99 ± 4.50	16.39 ± 2.33	0.808
Selenium (μg/d）	47.61 ± 11.49	59.06 ± 22.23	0.165
Copper (mg/d)	1.51 ± 0.47	1.46 ± 0.40	0.800
Manganese (mg/d)	2.06 ± 0.63	2.58 ± 0.72	0.103

Student's *t* test on normalized continuous variables, and Wilcoxon rank-sum test on non-normal continuous variables.

Table S6 Characteristics of patients in five CKD stages and HC

**Parameter**	Normal or high eGFR CKD (eGFR ≥ 90 mL/min/1.73m^2) **(n=10)**	Mild CKD (eGFR ≥ 60~89 mL/min/1.73m^2) **(n=10)**	Moderate CKD (30~59 mL/min/1.73m^2) **(n=10)**	Severe CKD (15~29 mL/min/1.73m^2) **(n=10)**	End stage CKD (<15 mL/min/1.73m^2) **(n=10)**	**HC (n=10)**	***P* value**
Female	5 (50.00)	5 (50.00)	5 (50.00)	5 (50.00)	5 (50.00)	5 (50.00)	1.000
Age (yrs)	59.9 ± 16.04	49.6 ± 20.33	51.5 ± 15.76	54.1 ± 17.53	61.8 ± 16.1	57.8 ± 11.76	>0.05
BMI	25.27 ± 3.43	24.33 ± 3.69	25.53 ± 4.72	24.72 ± 2.85	23.26 ± 3.62	24.72 ± 1.69	>0.05

A one-way ANOVA was used for comparing the nutrient intake among the groups.
